# Systematic review of the clinical effect of glucocorticoids on nonhematologic malignancy

**DOI:** 10.1186/1471-2407-8-84

**Published:** 2008-03-28

**Authors:** Bruce D Keith

**Affiliations:** 1 Northern Ontario School of Medicine, Sault Area Hospital, Sault Ste. Marie, Ontario, Canada

## Abstract

**Background:**

Glucocorticoids are often used in the treatment of nonhematologic malignancy. This review summarizes the clinical evidence of the effect of glucocorticoid therapy on nonhematologic malignancy.

**Methods:**

A systematic review of clinical studies of glucocorticoid therapy in patients with nonhematologic malignancy was undertaken. Only studies having endpoints of tumor response or tumor control or survival were included. PubMed, EMBASE, the Cochrane Register/Databases, conference proceedings (ASCO, AACR, ASTRO/ASTR, ESMO, ECCO) and other resources were used. Data was extracted using a standard form. There was quality assessment of each study. There was a narrative synthesis of information, with presentation of results in tables. Where appropriate, meta-analyses were performed using data from published reports and a fixed effect model.

**Results:**

Fifty four randomized controlled trials (RCTs), one meta-analysis, four phase l/ll trials and four case series met the eligibility criteria. Clinical trials of glucocorticoid monotherapy in breast and prostate cancer showed modest response rates. In advanced breast cancer meta-analyses, the addition of glucocorticoids to either chemotherapy or other endocrine therapy resulted in increased response rate, but not increased survival. In GI cancer, there was one RCT each of glucocorticoids vs. supportive care and chemotherapy +/- glucocorticoids; glucocorticoid effect was neutral. The only RCT found of chemotherapy +/- glucocorticoids, in which the glucocorticoid arm did worse, was in lung cancer. In glucocorticoid monotherapy, meta-analysis found that continuous high dose glucocorticoids had a detrimental effect on survival. The only other evidence, for a detrimental effect of glucocorticoid monotherapy, was in one of the two trials in lung cancer.

**Conclusion:**

Glucocorticoid monotherapy has some benefit in breast and prostate cancer. In advanced breast cancer, the addition of glucocorticoids to other therapy does not change the long term outcome. In GI cancer, glucocorticoids most likely have a neutral effect. High dose continuous glucocorticoids have a detrimental effect in nonhematologic malignancy. Glucocorticoid therapy might have a deleterious impact in lung cancer.

## Background

Glucocorticoids are frequently used in the treatment of nonhematologic malignancy to relieve symptoms of cancer and its treatment. For example, glucocorticoids prevent vomiting and allergic reactions associated with cancer therapy. Glucocorticoids decrease edema in CNS malignancy, and can decrease pain secondary to cancer.

Glucocorticoids are part of the treatment of some cancers. Glucocorticoids, as monotherapy and in combination with ketoconazole or chemotherapy, are used in prostate cancer. They are an option for postmenopausal women with breast cancer. Thymomas are another indication for glucocorticoids. Lymphoma and multiple myeloma can respond to glucocorticoids.

The effect of glucocorticoids on the treatment of solid tumors has been reviewed. In both reviews, the possibility, that combination therapy with glucocorticoids could be detrimental, was raised. In both reviews, there was no mention of prospective clinical studies [1,2].

Glucocorticoids are commonly used; the prospect of glucocorticoids having an unfavorable effect on cancer therapy has been brought up. With this in mind, a systematic review was done of clinical research concerning glucocorticoids in nonhematologic malignancy. The data regarding the effects of glucocorticoids, as monotherapy and in combination with other therapies (chemotherapy, hormonal therapy, radiotherapy, surgery), on nonhematologic malignancy was studied. The purpose is to make medical practice recommendations and suggest future research directions.

## Methods

All RCTs found of glucocorticoids in nonhematologic malignancy that looked at any of the endpoints of tumor response, tumor control (time to disease progression, time to treatment failure, progression free survival) or overall survival were included. A trial was considered randomized if it was described as such in the manuscript. All such trials that compared a glucocorticoid arm to a nonglucocorticoid arm or compared two glucocorticoid arms were included. All meta-analyses of such randomized controlled trials were included. All phase l/ll trials found of glucocorticoid monotherapy in nonhematologic malignancy, other than breast or prostate cancer, were included. All case series found of glucocorticoid monotherapy in nonhematologic malignancy, other than breast cancer or prostate cancer or thymoma, that contained tumor response data were included. All case reports in nonhematologic malignancy, that showed either tumor suppression or enhancement in response to glucocorticoid monotherapy, were included. In breast cancer, prostate cancer and thymoma, case reports of tumor shrinkage in response to glucocorticoid monotherapy were excluded. Trials, that were unpublished or published in abstract form only, were included.

A literature search was performed using the National Library of Medicine PubMed database (1950-September 25, 2007), EMBASE (1974–2007, week 38), Cochrane Central Register of Controlled Trials (to third quarter 2007), Cochrane Database of Systematic Reviews (to third quarter 2007), Cochrane Database of Abstracts of Reviews of Effects (to third quarter 2007), ACP Journal Club (1991-September/October 2007), Index Medicus (1949–1965), Excerpta Medica (1947–1979), CINAHL (1977-September Week 3 2007), and reference lists in published papers. See Additional file 1 for the search strategies of PubMed, EMBASE, the Cochrane Library (including ACP Journal Club) and CINAHL. The Related Articles feature of PubMed was used to search for additional articles. Science Citation Index was used to check for articles citing the publications making up the systematic review. Conference proceedings of AACR (1953–2007), ASCO (1974–2007), ASTRO/ASTR (1977–2006), ESMO (1977–2006), ECCO (1991–2005) and other relevant conferences were hand searched. The National Guidelines Clearinghouse was used to check for guidelines under the disease category neoplasms available as of September 24, 2007. The literature was searched for randomized controlled trials, meta-analyses, phase l/ll trials, other clinical observational studies, practice guidelines and reviews (systematic and nonsystematic). There was no language restriction on the literature search. For some trials, authors were contacted to obtain more information. Authors, who had published more recently in this field, were contacted to see if they were aware of any unpublished case series, clinical trials or meta-analyses. Textbooks of medical oncology, radiation oncology and palliative care were reviewed to obtain further references. Ongoing trials (as of September 27, 2007) were searched for at the Clinical Trials section of the NCI website (cancer.gov) using the drug names cortisone, dexamethasone, methylprednisolone, prednisolone, prednisone and therapeutic hydrocortisone.

The author determined the eligibility of the RCTs, meta-analysis, phase l/ll trials, case series and case reports that resulted from the search. Information, regarding trial design, patient characteristics, therapy, results and quality criteria were extracted from the eligible studies by the author. Data was extracted using a standard form.

For RCTs, the quality criteria published by Jaddad et al were used. In this instrument, 0 to 2 points are assigned for randomization, 0 to 2 points are assigned for double blinding and 0 to 1 points are assigned for the description of withdrawals and dropouts. This gives a score ranging from 0 to 5 with a higher score being better [3]. For phase l trials, phase ll trials and case series, the quality criteria are those described by the Centre for Reviews and Dissemination for case series [4]. No published quality criteria for meta-analyses were found, so one was devised.

Information from the clinical trials, case series and the meta-analysis is presented in tables, with the results being synthesized in a narrative manner.

Where RCTs were similar enough in patient characteristics, therapy and endpoints, meta-analyses were performed. Response definitions were those used by the authors of each RCT; only complete and partial responses were included as responses. Followup was assumed to be complete. Survival at selected time points was extrapolated from graphs. If survival graphs were not provided, exponential survival was assumed and the median survival was used to generate survival data. Within trials, the response rates and survival in the two arms formed 2 by 2 tables for which odds ratios and their corresponding 95% confidence intervals and between trial comparisons were made using logistic regression. Homogeneity of the odds ratios across trials was evaluated using a Breslow-Day χ^2 ^test. Odds ratio estimates and between-group comparisons for the combined trials were made using the Mantel-Haenszel fixed effect model. A result had to have a p value of less than 0.05 to be considered statistically significant.

## Results

### Results of Literature Search

The literature was searched for clinical studies of glucocorticoids. Studies were eligible if patients had nonhematologic malignancy, and if there were endpoints of tumor response or tumor control or survival. For RCTs and meta-analyses, trials comparing two glucocorticoid arms or a glucocorticoid arm to a nonglucocorticoid arm were eligible. After excluding duplicate publications of the same trial, fifty four RCTs met the eligibility criteria ([5-54] and [55-61]); one meta-analysis met the eligibility criteria [62]. In nonhematologic malignancy other than breast or prostate cancer, four phase l/ll (nonrandomized) trials of glucocorticoid monotherapy were found [63-66]. In nonhematologic malignancy other than breast cancer or prostate cancer or thymoma, four case series of glucocorticoid monotherapy with tumor response data were identified [67-70]. One of the case series is a clinical trial; however, tumor response rate was not a preplanned endpoint, so it is presented as a case series [67]. Three clinical trials, with no available results, were identified [71-73]. Nine case reports of glucocorticoid monotherapy, other than breast cancer or prostate cancer or thymoma regressing in response to glucocorticoid monotherapy, were found [74-83]. No article was excluded due to quality.

Glucocorticoids are commonly given as premedication with chemotherapy to prevent nausea and vomiting. In the chemotherapy trials mentioned in this review, there are two trials where it is clear whether glucocorticoids were used as premedication in patients not on the glucocorticoid arm [23,28]. In the other chemotherapy trials, there is no mention of whether such usage was permitted or not.

### Case Reports

There are case reports of nonhematologic malignancy either regressing or having increased growth in response to glucocorticoids. It is reasonably well documented in the literature that thymoma can sometimes respond to glucocorticoids [84]. In melanoma, 2 doses of 100 mg of intravenous hydrocortisone 8 hours apart caused tumor lysis syndrome in a patient with melanoma [74]. There are case reports where glucocorticoids apparently stimulated the growth of melanoma [75,76]. There is a case report of liver metastasis of thymic carcinoid responding to prednisolone [77]. Metastatic renal cell carcinoma might have shown a complete response to glucocorticoids in two patients[78,79]. There are case reports where glucocorticoids apparently stimulated the growth of breast cancers [80-83].

### Case Series

The four case series of glucocorticoid monotherapy are shown in Table 1[67-70]. They included patients with a variety of cancers, such as lung cancer, GI cancer, breast cancer and sarcoma. No tumor regression, objective improvement or relief of pain was noted. Glucocorticoid doses were not dissimilar taking into account relative glucocorticoid activities (Table 2). For any particular tumor type, the number of cases is small: the one exception is the 255 lung cancer patients in the case series of de Camp [69].

**Table 1:** Case Series of Glucocorticoid Monotherapy

Author [reference], Year	Patient Characteristics	Treatment	Responses
Postlethwait et al [68], 1951	4 gastric, 1 rectal, 1 gallbladder, 1 esophageal, all advanced stage	300 mg IM of cortisone on day 1, 200 mg IM on day 2 then 100 mg IM daily for 21 days; one patient received 150 mg instead of 100 mg	no objective improvement
de Camp [69], 1961	255 bronchogenic carcinoma, 19 primary lung tumors of other origins (sarcoma, adenomatosis etc.), 6 alveolar cell carcinoma of lung, 14 with cancer metastatic to lung, 26 pleural carcinoma, 2 pericardial carcinoma	patients received 30–40 mg of prednisone/prednisolone per day, which was tapered to 10–15 mg per day (occasionally to 5 mg per day) or 4 mg of dexamethasone per day tapered to 1–1.5 mg per day	no effect, either positive or negative
Plengvanit and Viranuvatti [70], 1964	7 primary carcinoma of the liver	40–60 mg of daily prednisone for 2 weeks to 10 months	little value as based on effect on pain and liver size (by physical examination)
Bruera et al [67], 1985	8 colon, 7 breast, 4 lung, 4 soft tissue sarcoma, 2 kidney, 2 prostate, 1 head and neck, 1 melanoma, 1 pancreas, 1 ovarian	methyprednisolone 16 mg po twice daily for 25 out of 34 days	no tumor regression

**Table 2:** Relative Glucocorticoid Activities of Glucocorticoids

	Glucocorticoid Activity
Cortisol (Hydrocortisone)	1
Cortisone	0.8
Prednisone	4
Prednisolone	4
Methylprednisolone	5
Dexamethasone	30

With regards to quality, two case series were from tertiary care centers; the two that were not originated from secondary care institutions [67,69]. Three of the four case series had less than 50 patients [67,68,70]. None of the case series were multi-institutional. Three of the four case series did not describe eligibility criteria [68-70]. In one case series, patients were similar with regards to the state of cancer progression; in the other studies, not enough information was provided to assess similarity [67]. In one case series, followup was long enough to assess whether glucocorticoids could cause cancer to decrease in size; in the other studies, it was unclear if followup was sufficient to assess this [70]. In none of the case series was reduction in tumor size assessed using objective criteria.

### Phase l and Phase ll (Nonrandomized) Trials

In phase l/ll (nonrandomized) trials, responses have been noted. Based on responses lasting at least 28 days, responses in the four trials were 0% [63], 6% [64], 6% [65] and 6% [66]. In the largest trial (407 patients), 13 of the 24 responses were in breast and prostate cancer with the 2 complete responses being in breast and prostate cancer [66]. The next largest trial was 94 patients with no responses [63]. The phase l/ll (nonrandomized) clinical trials are presented in Table 3.

**Table 3:** Glucocorticoid Monotherapy Activity in Phase l and Phase ll (Nonrandomized) Trials

Author [reference], Year	Patient Characteristics	Treatment	Responses
Mass [63], 1964	32 lung cancer (17 epidermoid, 7 adenocarcinoma, 8 undifferentiated) 26 GI, 18 GU, 7 melanoma, 2 breast	25–100 mg flurometholone po per day for 7 weeks followed by taper	None
Moertel et al [64], 1964	18 colon, 13 gastric, 9 pancreatic, 5 carcinoid, 12 primary unknown (presumed GI), 6 miscellaneous GI, 1 renal cell	25 mg fluorometholone po every 12 hr for at least 2 months	4 PR (1 colon, 1 gastric, 2 primary unknown)
Johnson et al [65], 1966	44 melanoma, 9 lung carcinoma, 5 ovary, 4 uterus, 4 prostate, 4 kidney, 4 breast, 7 miscellaneous	200–600 mg of NSC-17256 per day po for 8 weeks	melanoma (3 CR, 2 PR)
Ramirez et al [66], 1971	24 head and neck, 1 gastric, 36 colorectal, 2 pancreas, 13 lung, 70 breast, 36 cervix, 12 uterus, 9 ovary, 9 prostate, 27 kidney, 2 bladder, 111 melanoma, 1 thyroid, 6 liver, 29 sarcoma, 11 primary unknown, 1 lymphoma, 7 miscellaneous	200–600 mg of NSC-17256 per day po for 6 weeks; average dose 300 mg per day	Head and neck (1 PR), breast (1 CR, 8 PR), uterus/cervix (2 PR), ovary (1 PR), prostate (1 CR, 3 PR), melanoma (6 PR), Hodgkin (1 PR)

The two glucocorticoids used in these clinical trials were fluorometholone and NSC-17256. Fluorometholone may have progestational properties [22]. One hundred mg of NSC-17256 is equivalent to 50–67 mg of prednisone; however, it also has sex steroid properties.

With regards to quality, all the clinical trials were from tertiary care centers. All clinical trials had more than 50 patients. Three of the four trials were multi-institutional [63,65,66]. One of the trials did not describe eligibility criteria [65]. In one trial, patients were similar with regards to the state of cancer progression; in the other trials, not enough information was provided to assess similarity [64]. In two trials, followup was long enough to assess whether glucocorticoids could cause cancer to regress in size; in the other two trials, it was unclear if followup was sufficient to assess this [64,66]. Reduction in tumor size was assessed using objective criteria in three of the studies [64-66]; this was unclear in the other study.

### Randomized Controlled Trials of Glucocorticoids in the Endocrine Therapy of Advanced Breast Cancer

There are two types of trials in this category. In the first type, glucocorticoids are given as monotherapy and compared to other endocrine therapies given as monotherapy. There are ten trials of this type, which tend to be small and often use endocrine therapies that are no longer given. In these trials, the vast majority of patients were postmenopausal or enrolment was limited to postmenopausal patients. The results show that glucocorticoids have modest activity in postmenopausal women. A description of these trials is given in Table 4[5,15,18,19,22,38,47,55,56]. The three earliest trials used response criteria that are not readily comparable to presently used response criteria [5,19,22].

**Table 4:** Randomized Controlled Trials of Glucocorticoids in the Endocrine Therapy of Advanced Breast Cancer

Author [reference], Year	Patient Characteristics	Treatment Arms and Patient Numbers (Randomized/Evaluable)	Quality Score	Outcome
Dao et al [19], 1961	postmenopausal, previous androgens or estrogens for advanced disease	bilateral adrenalectomy + hormone replacement (19/18) vs. cortisone acetate (at least 3 months; 300 mg on first day tapered to 50 mg daily) (20/19)	1	8 vs. 0 remissions
Colsky et al [5], 1963	Postmenopausal	100 mg of 9α-bromo-11β-ketoprogesterone po every 8 hrs for at least 60 days (32/23) vs. same except 13.2 mg of prednisolone (23/18)	4	0 vs. 1 remission
Talley et al [22], 1964	postmenopausal, no previous endocrine therapy	10 mg of fluoxymesterone (an androgen) po twice daily (23/21) vs. 25 mg of oxylone acetate po twice daily (23/22)	2	3 vs. 6 remissions (ns)
Talley et al [22], 1964	postmenopausal, previous androgens or estrogens	25 mg of oxylone acetate po twice daily (14/NR) vs. 12 mg of methylprednisolone twice daily (13/NR)	4	3 vs. 3 remissions
Gaertner et al [18], 1968	Postmenopausal	dromostanolone propionate (an androgen) 100 mg IM 3 times weekly (22/NR) vs. fluorometholone 25 mg po daily (22/NR) vs both together (24/NR)	3	9 vs. 2 vs. 5 responses
Goldenberg [15], 1969	postmenopausal, no previous hormonal therapy	Testololactone (an androgen) 75 mg twice daily po (103/100) vs. MPA 50 mg twice daily po (108/104) vs. oxylone acetate 25 mg twice daily po (108/107)	2	response rates of 4.9% vs. 9.3% vs. 19.4%; p = 0.052 for MPA and oxylone being equal
Jakobsen et al [38], 1986	95% previous antiestrogen therapy, 83% postmenopausal	prednisone 10 mg po three times daily (52/43) vs. MPA 500 mg po daily (48/38) vs. MPA 100 mg IM daily except Sat/Sun for 3 weeks then 500 mg IM weekly(50/40); continued until PD	1	response rates of 4.6% vs. 7.9% vs. 12.5% (ns), median time to progression of 3 vs. 2.5 vs. 4 months (p = 0.09), median survival of 6 vs. 8.5 vs. 10 months (p = 0.30)
Wander et al [56], 1987	81% postmenopausal, 40.5% ER and/or PgR +ve, 26.7% ER/PgR -ve, 32.8% ER/PgR unknown	aminoglutethimide 1000 mg po daily + cortisone acetate 50 mg po daily (65/62) vs. aminoglutethimide 1000 mg po daily + MPA 1000 mg po daily (73/69)	1	response rates (CR/PR) of 6.5%/25.8% vs. 7.3%/24.6%
Kristensen et al, [47] 1992	Hypercalcemia due to breast cancer	standardized isotonic saline + IV furosemide (15/15) vs. same + prednisolone 25 mg po three times daily for 8 days (15/15)	3	median survival of 40 days vs. 84 days (p = 0.46)
Mercer et al, [55] 1993	postmenopausal; PD on tamoxifen (adjuvant or advanced setting)	aminoglutethimide 125 mg twice daily (28/27) vs. hydrocortisone 20 mg twice daily (33/29)	2	response rates in 11% vs. 21% (p > 0.1), time to treatment failure similar (p = 0.743), overall survival similar (p = 0.240)
Stewart et al [24], 1982	Only previous systemic therapy allowed was adjuvant chemotherapy, 98 ER +ve, 28 ER -ve, 54 ER unknown	Premenopausal: ovarian irradiation (19) vs. same + prednisolone 5 mg twice daily (16) Postmenopausal: tamoxifen 10 mg twice daily (72) vs. same + prednisolone 5 mg twice daily (73); treatment until PD; 204 randomized, 180 evaluable	2	Premenopausal/Postmenopausal response rates: 21% vs. 44% (ns)/13% vs. 36% responses (p < 0.01), median survival 19 vs. 18 months (ns)/12 vs. 21 months (p < 0.025)
Rubens et al [46], 1988	Only previous systemic therapy allowed was adjuvant chemotherapy (15%): ER/PgR unknown (23%) or ER+ve or PgR+ve	Premenopausal: ovarian irradiation with prednisolone 5 mg twice daily added on progression (NR/15) vs. ovarian irradiation + prednisolone 5 mg twice daily until PD (NR/16)/Postmenopausal: tamoxifen 10 mg twice daily changed to prednisolone 5 mg twice daily on progression (NR/78) vs. tamoxifen 10 mg twice daily +prednisolone 5 mg twice daily until PD (NR/85); 220 randomized/194 evaluable	2	Premenopausal/Postmenopausal response rates: 27% vs. 63% (p < 0.05)/31%vs46%(p < 0.1), median time to disease progression: 4 vs. 14 months (p = 0.006)/4 vs. 8 months (p = 0.02), median survival: 17 vs. 66 months (p = 0.04)/17.5 vs. 21 months (p = 0.3)
Ingle et al, [7] 1991	postmenopausal; ER/PR unknown (15%) or ER+ve or PgR+ve; only previous systemic therapy allowed was adjuvant chemotherapy (7.8%)	tamoxifen 10 mg po twice daily (162/159) vs. tamoxifen 10 mg po twice daily + prednisolone 5 mg po twice daily (164/161); continued until PD	5	responses in 38% vs. 47% (p = 0.15), median time to progression: 11 vs. 10 months (p = 0.81), median survival: 35 vs. 32 months (p = 0.40)
Cocconi et al, [37] 1992	postmenopausal; ER/PgR unknown (48%) or ER+ve or PgR+ve; no previous endocrine therapy for advanced disease; previous chemotherapy or endocrine therapy in 39%	aminoglutethimide 125 mg po twice daily for 1 month then 250 mg po twice daily (84/78) vs. same + hydrocortisone 20 mg po twice daily (87/83); treatment until PD	3	responses in 41% vs. 44% (ns), median time to progression: 6.3 vs. 8.1 months (p = 0.38), median survival: 36.3 vs. 34.2 months (p = 0.56)

**Figure 1:**
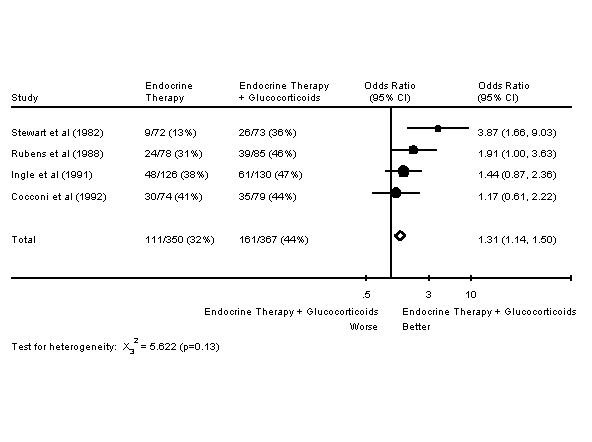
Forest Plot of Response Rates of Endocrine Therapy +/- Glucocorticoids in Advanced Breast Cancer.

**Figure 2:**
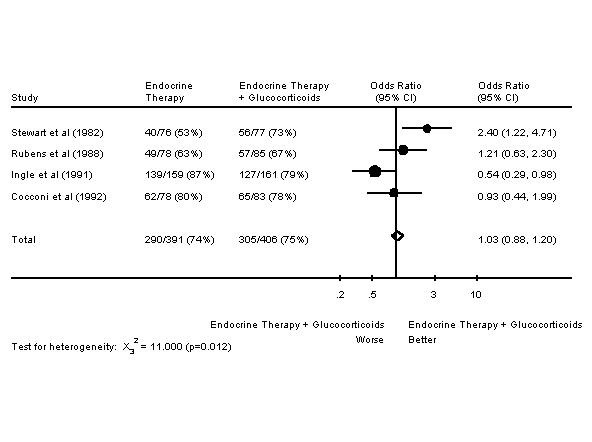
Forest Plot of One Year Survival Rates of Endocrine Therapy +/- Glucocorticoids in Advanced Breast Cancer.

The second type of trial is of an endocrine agent +/- glucocorticoids. These trials tend to be larger, more recent and limited to women who are postmenopausal or who received ovarian irradiation. There are four trials in this category: three are of tamoxifen +/- glucocorticoids and one is of aminoglutethimide +/- glucocorticoids. Results are presented in Table 4[7,24,37,46]. As these trials were more similar in nature, meta-analyses of response rates and survival were undertaken. As seen in Figure 1, the addition of glucocorticoids to another endocrine therapy resulted in an increased response rate. Figure 2 shows that this addition does not change one year survival rates. However, there was evidence for a lack of homogeneity among the four studies in Figure 2, as shown by the chi-square test and its p value. The meta-analysis was repeated, except that the trial which was furthest from the other three trials in response rate and survival was omitted [24]. The result of the omission was that the addition of glucocorticoids did not change one year survival rates, although the lack of homogeneity disappeared (odds ratio of 0.91 95% CI 0.77–1.08, X_2_^2 ^= 3.389 p = 0.184).

One unpublished trial of hormonal therapy +/- glucocorticoids, with no available results, was found [72].

### Randomized Controlled Trials of Glucocorticoids in the Endocrine Therapy of Breast Cancer in the Adjuvant Setting

In this setting, there are trials of endocrine therapy +/- glucocorticoids and trials of glucocorticoid monotherapy compared to no therapy: they are summarized in Table 5. The trials of endocrine therapy +/- glucocorticoids consist of two trials of tamoxifen +/- glucocorticoids in postmenopausal women; these trials are negative [6,9]. This is consistent with the results from the advanced breast cancer setting.

**Table 5:** Randomized Controlled Trials of Glucocorticoids in the Endocrine Therapy of Breast Cancer in the Adjuvant Setting

Author [reference], Year	Patient Characteristics	Treatment Arms; Patient Number (Randomized/Evaluable)	Quality Score	Outcome(s)
DiMartino et al [9], 1991	Postmenopausal	Tamoxifen 40 mg per day vs. same + prednisolone 7.5 mg per day; 67/57 vs. 49/47; treatment until recurrence	1	p values of 0.6743 and 0.2367 for differences in disease free survival and overall survival respectively
Fentiman et al [6], 1994	Postmenopausal	Tamoxifen 20 mg daily vs. same + prednisolone 7.5 mg daily; 186/173 vs. 184/168	2	p values of 0.26 and 0.32 for differences in relapse free survival and overall survival respectively
Meakin et al, [44], 1996		no further treatment (NT) vs. ovarian irradiation (R) vs. (if ≥ 45) ovarian irradiation + prednisolone 7.5 mg daily up to 5 years (R +P); Premenopausal (≥ 45 years old) 64 vs. 71 vs. 73/Postmenopausal 136 vs. 111 vs. 111; 779 randomized, 703 analyzed as above	1	Premenopausal(≥ 45 years old)/Postmenopausal; median recurrence free survival (yrs): 9.4 vs. 18 vs. > 25/6.2 vs. 8.2 vs. 6.8; median survival (yrs): 12.8 vs. 14.9 vs. >25/9.3 vs. 9.9 vs. 7.7
Scottish Cancer Trials Breast Group [13], 1993	Premenopausal ER+ve 54%, ER status unknown 19%	Second randomization to prednisolone 7.5 mg po daily for 5 years vs. no prednisolone after first randomization to ovarian ablation vs. CMF (IV); 165/NR vs. 167/NR in second randomization	3	prednisolone effect did not depend on whether CMF or ovarian ablation (p = 0.46); hazard ratio and 95% CI for deaths for +/-prednisolone was 1.26 (0.86–1.84)

In the first of the two trials of glucocorticoid monotherapy, glucocorticoids weren't beneficial in the postmenopausal group; they might have been beneficial in the premenopausal group, who were treated with ovarian irradiation [44]. In the second trial of glucocorticoid monotherapy, premenopausal women were randomly treated with chemotherapy or ovarian ablation followed by a second randomization to glucocorticoids versus no glucocorticoids. Regardless of whether a woman received chemotherapy or ovarian ablation, glucocorticoids were not beneficial. In the 81% of patient in whom ER status was known, chemotherapy tended to be more effective than ovarian ablation in those with low ER concentrations whereas the opposite was true in those with higher ER concentrations. No such relationship was noted when the no prednisolone/prednisolone arms were compared [13].

One feature that complicates interpretation of the second trial is that glucocorticoids were started at the time of oophorectomy or at the start of chemotherapy, and were to be given for 5 years unless relapse occurred. Those in the chemotherapy arm got glucocorticoids during their 24 weeks of chemotherapy. This means that this trial could also be considered a chemotherapy +/- glucocorticoids trial. Glucocorticoid administration resulted in less bone marrow suppression during chemotherapy [13].

### Randomized Controlled Trials of Chemotherapy +/- Glucocorticoids in Advanced Breast Cancer

There are eight trials of chemotherapy +/- glucocorticoids in advanced breast cancer [16,17,21,25,28,31,40,48]; results are presented in Table 6. Decreased thrombocytopenia [21,40] and increased administered chemotherapy dose [21,40,48] were associated with glucocorticoid administration in several of these trials.

**Table 6:** Randomized Controlled Trials of Chemotherapy +/- Glucocorticoids in Advanced Breast Cancer

Author [reference], Year	Patient Characteristics	Treatment Arms Patient Number (Randomized/Evaluable)	Quality Score	Outcome(s)
Brambilla et al [17], 1974	Postmenopausal	adriamycin 40–75 IV mg/m^2 ^day 1, vincristine IV 1.4 mg/m^2 ^days 1 and 8 every 3 weeks for 8 cycles (20/15) vs same + prednisone 100 po mg/m^2^/day days 1–5 (22/21)	2	response rates of 50% vs. 55%
Ramirez et al [16], 1975		5-fluorouracil (5-FU) 10 mg/kg/week IV, methotrexate 0.5 mg/kg/week IV, vincristine 0.02 mg/kg/week IV, cyclophosphamide 2 mg/kg/day po (NR/52) vs. same + prednisone po 45 mg/day for 2 weeks, then 30 mg/day for 2 weeks then 15 mg/day (NR/48); treatment until PD	2	response rates of 44.2% vs. 62.5% (p = 0.075), no significant difference in response duration or survival
Rossi et al [25], 1976	Pre or postmenopausal, no previous chemotherapy	L-phenylalanine 4 mg/m^2 ^po/day + 5-FU 300 mg/m^2 ^po/day each days 1–5 every 28 days (16/14) vs. same except 5-FU IV (18/18) vs. same as first except 5-FU IV + prednisone 30 mg/m^2^/day days 1–5 (17/15)	1	response rates of 7% vs. 22% vs. 40% (ns)
Tormey et al [21], 1982	Pre or postmenopausal, no previous chemotherapy or prednisone therapy	CMF (NR/79) vs. CMFP (NR/86) vs. AV (NR/166); after 6 months or if progressive disease prior to then, switched to nonglucocorticoid containing chemotherapy	1	response rates of 57% vs. 63% vs. 56% (p > 0.10), response durations of 4.5 months vs. 8.4 months vs. 7.7 months (p = 0.05), time to treatment failure of 5.3 months vs. 9.1 months vs. 5.7 months (p = 0.04), overall survival of 14.5 months vs. 16.4 months vs. 13.7 months (p = 0.03)
Tormey et al [48], 1983	Pre or postmenopausal, no previous chemotherapy or prednisone therapy	CMF (NR/47) vs. CMFP (NR/47) vs. rotation every 2 cycles between CMF and AV (NR/50); treatment for 6 months followed by nonglucocorticoid containing therapy	2	response rates of 55% vs. 70% vs. 58% (ns), response duration of CMF less than CMFP (p = 0.39); time to treatment failure of CMF 5.6 months and CMFP 7.0 months (p = 0.16); median survival of CMF 12.5 months and CMFP 18.0 months
Gercovich et al [31], 1989	Untreated	5-FU + cyclophosphamide + mitoxantrone vs. same + prednisone 80 mg po days 1–5 (53/50 in total)	1	no difference in response rates
Tashiro & Nomura [40], 1995	resistant to or relapsed after adriamycin treatment, pre or postmenopausal	mitomycin C 4 mg/m^2 ^+ methotrexate 35 mg/m^2 ^IV + vincristine 0.7 mg/m^2 ^IV days 1 and 8 every 21 days (21/21) (MMV) vs. same + prednisolone 10 mg po per day (MMVP) (43/41) vs. same as first + MPA 1200 mg po per day (MMVM) (44/40); treatment until PD	2	response rates of 9.5% vs. 29% vs. 37.5% (p = 0.0784 comparing MMV to MMVP); median duration of response of 12 vs. 16 vs. 34 weeks (ns); time to progression for MMVM longer than MMVP which was longer than MMV (p = 0.0256); survival differences (p = 0.382)
Piccart et al [28], 1997	One previous chemotherapy regimen for advanced or metastatic breast cancer	docetaxel 50 mg/m^2 ^IV days 1 and 8 every 3 weeks (42/41) vs. same + methylprednisolone 40 mg po on days -1,0,1,7,8 and 9 (41/39)	2	no significant differences in median response duration, median time to progression or median survival

**Figure 3:**
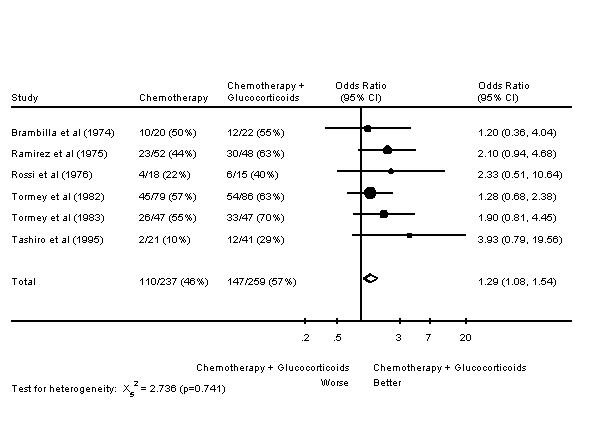
Forest Plot of Response Rates of Chemotherapy +/- Glucocorticoids in Advanced Breast Cancer.

**Figure 4:**
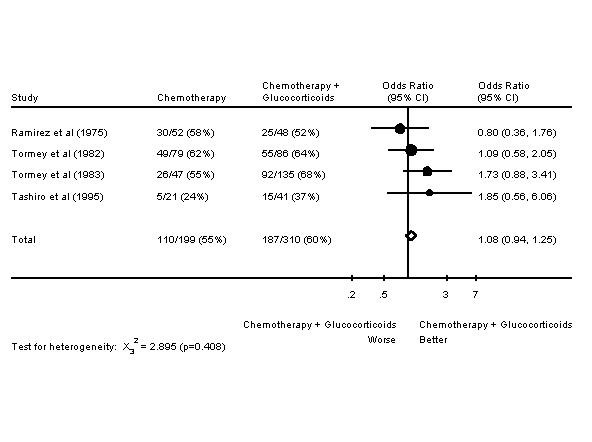
Forest Plot of One Year Survival Rates of Chemotherapy +/- Glucocorticoids in Advanced Breast Cancer.

In six of these trials, response rates are given; in four of these trials, survival data is given. It was felt that there was enough similarity in these trials to perform meta-analyses. Meta-analyses of response rates and survival are presented in Figures 3 and 4 respectively. The addition of glucocorticoids to chemotherapy in the advanced breast cancer setting resulted in an increased response rate. However, there was no effect on one year survival.

### Randomized Controlled Trials of Chemotherapy +/- Glucocorticoids of Breast Cancer in the Adjuvant Setting

There are two trials in this category. Both are relatively large trials of CMF+/-prednisone, and both are negative. This is consistent with what has been found in the advanced breast cancer setting. At the time of enrolment, none of the participants were postmenopausal. In comparison to trials that enroll postmenopausal women, this might lessen the possible impact of glucocorticoids as endocrine therapy. However, many women on these trials subsequently developed amenorrhea. In the trial of Tormey et al, 20% subsequently became postmenopausal. In the trial of the Ludwig Breast Cancer Study Group, 85% subsequently became postmenopausal. A description of these trials is given in Table 7[12,53,85].

**Table 7:** Randomized Controlled Trials of Chemotherapy +/- Glucocorticoids of Breast Cancer in the Adjuvant Setting

Author [reference], Year	Patient Characteristics	Treatment Arms Patient Number (Randomized/Evaluable)	Quality Score	Outcome(s)
Tormey et al [12], 1990	Premenopausal, ER+ve 51%, ER-ve 49%,	CMF (222/188) vs. CMFP (220/183) vs. CMFP + tamoxifen 10 mg po twice daily(220/182), all treatments for one year	2	median time to relapse: CMF 6.2 months vs. CMFP 6.6 months (p = 0.64), survival: CMF vs. CMFP (p = 0.91)
Ludwig Breast Cancer Study Group [53, 85], 1985	pre and peri-menopausal, ER +ve 28%, ER -ve 24% ER unknown 48%	CMF (250/241) vs. CMF + prednisone 7.5 mg daily (255/250), all treatments for one year	3	13 year disease-free survival of 52% vs. 49% (p = 0.39), 13 year overall survival of 65% vs. 59% (p = 0.30)

As in the randomized controlled trials of chemotherapy +/- glucocorticoids in the advanced breast cancer setting, there is evidence of decreased leukopenia [53], decreased thrombocytopenia [12], increased chemotherapy dose [53], and increased chemotherapy dose intensity [12] in the glucocorticoid arms.

In one of the trials, there was a significant increase in bone, alone or in combination with other sites, as the site of first relapse in the chemotherapy plus prednisone arm (relative risk of 2.06, 95% CI of 0.91 to 12.31, p = 0.09). The authors postulated that the increased rate of bone metastases as a first relapse site in this arm might be due to cytokine inhibition, which might reduce a putative anti-cancer process in bone, or to increased bone absorption [53].

### Randomized Controlled Trials of Glucocorticoids in Prostate Cancer

In prostate cancer, there are two RCTs of glucocorticoids in untreated patients [43,59]. Both are trials of orchiectomy versus orchiectomy plus glucocorticoids versus orchiectomy plus cyproterone acetate. The small numbers enrolled preclude analysis, other than it is improbable that glucocorticoids worsen outcome when added to orchiectomy. Both trials used response criteria that are not readily comparable to presently used response criteria.

There are five randomized controlled trials of glucocorticoids in patients with hormone refractory prostate cancer [8,32,33,35,36,54,60]. These are all trials comparing glucocorticoid monotherapy to other monotherapies. When glucorticoids are compared to other hormonal therapies (progestational agents and flutamide), little difference was noted. When glucocorticoids were compared to liarozole (a retinoic acid stimulating agent), glucocorticoids resulted in a better outcome. In the three oldest trials, the response criteria used are not readily comparable to presently used response criteria [8,33,35].

There is one randomized controlled trial of chemotherapy +/- glucocorticoids in prostate cancer [50]. This small trial has been published in abstract form only; it is difficult to draw conclusions based on the information presented.

See Table 8 for a description of these trials.

**Table 8:** Randomized Controlled Trials of Glucocorticoids in Prostate Cancer

Author [reference], Year	Patient Characteristics	Treatment Arms Patient Number (Randomized/Evaluable)	Quality Score	Outcome(s)
Sander et al [43], 1982	Advanced untreated prostate cancer	orchiectomy (7/7) vs. ochiectomy + prednisone 2.5 mg po four times daily for 6 weeks (14/14) vs. ochiectomy + cyproterone acetate 50 mg po four times daily for 6 weeks (13/13)	1	response rates of 86% vs. 93% vs. 62%
Williams et al [59], 1990	Advanced untreated prostate cancer	orchiectomy (24/24) vs. orchiectomy + dexamethasone 0.5 mg in morning and 0.3 mg at night (16/16) vs. orchiectomy + cyproterone acetate 100 mg 3 times daily (20/20)	3	response rates of 33% vs. 62.5% vs. 45% at 1 year
Fossa et al [33], 1985	Hormone refractory prostate cancer	MPA 500 mg po twice daily (24/21) vs. prednisolone 5 mg po four times daily (24/24)	2	response rates of 38% vs. 13% (p < 0.05), no survival difference
Patel et al [8], 1990	Hormone refractory metastatic prostate cancer	Megestrol acetate 40 mg po four times daily (29/25) vs. dexamethasone 0.75 mg po twice daily (29/26), Treatment until PD	2	response rates of 10% vs. 7%, median survival of 268 vs. 246 days (p = 0.2)
Datta et al [35], 1997	Hormone refractory metastatic prostate cancer	Flutamide 250 mg three times daily (20/20) vs. prednisolone 5 mg twice daily (20/20)	2	PSA response rates of 50% vs. 55%, median survival of 30 vs. 36 weeks (p > 0.05)
Tyrrell [36, 54, 60], 1996	Hormone refractory advanced prostate cancer	prednisone 10 mg po twice daily (103/NR) vs. liarozole 300 mg po twice daily (117/NR)	1	PSA response rates of 25% vs. 18% (p = 0.31); time to PSA progression of 4.7 vs. 3.5 months; median survivals of 15.8 months vs. 11.7 months (p = 0.01)
Fossa et al [32], 2001	Hormone refractory metastatic prostate cancer	prednisone 5 mg po four times a day (101/95) vs. flutamide 250 mg po three times a day (100/96); treatment until PD	2	PSA responses of 21% vs. 23%, median time to progression of 3.4 months vs. 2.3 months (p = 0.0885); overall survival of 10.6 months vs. 11.2 months (p = 0.8370)
Tombal et al [50], 2003	Hormone refractory metastatic prostate cancer	irofulven (24 mg/m^2 ^for first 18 patients then 0.55 mg/kg for next 43 patients) IV days 1 and 15 q4 weeks (29/28) vs. same + prednisone 10 mg daily (32/28)	1	PSA responses of 3/28 vs. 5/28

There are two ongoing randomized controlled trials of chemotherapy +/- glucocorticoids in hormone refractory prostate cancer; results are not available [71,73].

### Randomized Controlled Trials of Glucocorticoids in GI Cancer

There is one trial comparing glucocorticoid monotherapy to placebo in GI cancer; no difference in survival was found [26].

There are two trials of chemotherapy +/- glucocorticoids in GI cancer [11,20]. One of the two trials used response criteria that are not readily comparable to those presently used. In that trial, fluorometholone monotherapy had a response rate of 9%. The addition of fluorometholone to 5-FU did not change the response rate of 13% [20]. In the other trial of chemotherapy +/- glucocorticoids (FUDR +/- dexamethasone), the addition of glucocorticoids resulted in an increased response rate and a borderline improvement in survival (p = 0.06). However, FUDR dose, as the mean percentage of planned FUDR dose, was 61% and 52% in the FUDR/dexamethasone arm and FUDR arm respectively (p = 0.13). At least in part, the increased dose of FUDR in the FUDR/dexamethasone arm was due to the decreased biliary toxicity associated with concurrent dexamethasone administration resulting in less FUDR dose reductions. The chemoprotective property of dexamethasone, allowing a greater FUDR dose, may have contributed to the improved results in the combined arm [11]. Other postulated reasons are a potentiation of FUDR cytotoxicity by dexamethasone and a potential antiangiogenic property of dexamethasone [86,87].

There are two trials of preoperative glucocorticoids in patients scheduled to undergo esophagectomy; there was no effect on survival [51,58].

A description of the GI trials is given in Table 9.

**Table 9:** Randomized Controlled Trials of Glucocorticoids in GI Cancer

Author [reference], Year	Patient Characteristics	Treatment Arms Patient Number (Randomized/Evaluable)	Quality Score	Outcome(s)
Moertel et al [26], 1974	GI cancer with expected survival of less than 2 months (61 colorectal, 22 gastric, 15 pancreatic, 9 hepatoma, 10 other)	placebo (47/NR) vs. dexamethasone 0.75 mg four times daily (33/NR) vs. dexamethasone 1.5 mg four times daily (36/NR); treatment until death or patient unable to take pills	3	Median survival (weeks) of 6.6 vs. 6.2 vs. 5.2
Reitemeier et al [20], 1967	advanced GI cancer (37 gastric, 23 pancreatic, 52 unknown but presumed GI in origin)	fluorometholone 25 mg po every 12 hour for at least 2 months (34/32) vs. 5-FU IV 15 mg/kg/day for 5 consecutive days then 7.5 mg/kg/day every other day for a maximum of 4 additional doses (40/40) vs. 5-FU plus fluorometholone as just described (38/32)	3	response rates of 9% vs. 13% vs. 13%; average duration of response of 4 vs. 6.5 vs. 4 months
Kemeny et al [11], 1992	untreated colorectal cancer with only metastases being in liver	hepatic arterial FUDR 0.3 mg/kg/day for 14 of every 28 day cycle (25/25) vs. same plus hepatic arterial dexamethasone 20 mg over 14 days with the FUDR (25/24)	3	response rates of 40% vs. 71% (p = 0.03), median time to progression of 12 vs. 19 months (p = 0.58), median survival of 15 vs. 23 months (p = 0.06)
Sato et al [51], 2002	patients with esophageal squamous cell carcinoma scheduled to undergo esophageal resection	surgery (33/33) vs. surgery + 10 mg/kg methyprednisolone IV within 30 minutes of start of surgery (33/33)	5	p = 0.4465 for difference in overall survival rates 1 and 3 yr survival rates of 85% and 65% vs. 82% and 62% respectively
Yano et al [58], 2005	patients with thoracic esophageal cancer scheduled for esophagectomy	surgery (20/20) vs. surgery + methylprednisolone 500 mg IV 2 hours before surgery (20/20)	3	p = 0.3304 for difference in survival rates

### Randomized Controlled Trial of Glucocorticoids in Patients with Primary CNS Neoplasms

The one RCT of glucocorticoids in primary CNS neoplasms is described in Table 10. This trial uses a higher dose of glucocorticoids than any other trial described in this review; however, administration was on an intermittent, rather than a continuous, basis. This trial compared methyprednisolone versus BCNU versus BCNU plus methylprednisolone versus procarbazine. The addition of methylprednisolone to BCNU had a neutral effect on survival. Infection was significantly greater in the BCNU plus methylprednisolone arm than the other arms; information on the type and severity of infection is not available. The mean number of courses of chemotherapy was identical in the BCNU and BCNU + methylprednisolone arms. The mean radiotherapy dose +/- SE (rads) was 5701 +/- 79 in the methylprednisolone arm and 5589 +/- 96 in the BCNU + methylprednisolone arm [23].

**Table 10:** Randomized Controlled Trial of Glucocorticoids in Primary CNS Neoplasms

Author [reference], Year	Patient Characteristics	Treatment Arms Patient Number (Randomized/Evaluable)	Quality Score	Outcome(s)
Green et al [23], 1983	after surgery for supratentorial malignant glioma; 6000 rads of radiation to start concurrently with medical therapy	BCNU 80 mg/m^2^/day IV for 3 consecutive days every 8 weeks (147/124) vs. methylprednisolone 400 mg/m^2^/day orally for 7 consecutive days every 4 weeks (156/141) vs. BCNU + methylprednisolone as previously described (153/134) vs. procarbazine 150 mg/m^2^/day orally for 28 consecutive days every 8 weeks (153/128)	2	24 month survivals of 19.5% vs. 8.0% vs. 18.0% vs. 22.2%; in pairwise comparisons, only differences giving p < 0.05 were BCNU vs. methylprednisolone and procarbazine vs. methylprednisolone

### Randomized Controlled Trials of Glucocorticoids in Patients with GU Neoplasms

No trials were found in this category.

### Randomized Controlled Trials and Meta-analysis of Glucocorticoids in Patients with a Variety of Cancers

There are five randomized controlled trials of glucocorticoid monotherapy in patients with a variety of cancers; these trials are dissimilar in nature.

**Figure 5:**
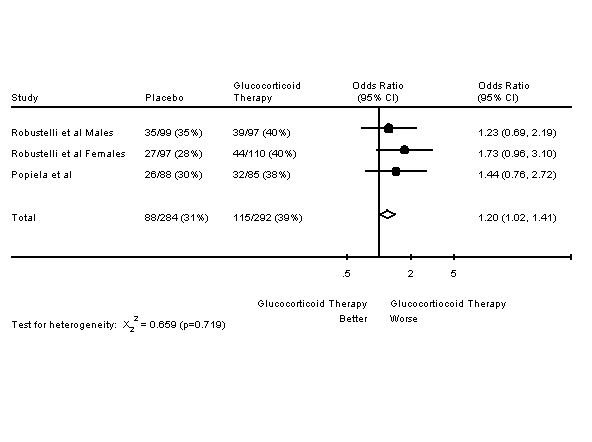
Forest Plot of Eight Week Mortality Rates in Two Trials of Glucocorticoid Monotherapy in Nonhematologic Malignancy.

The first two trials used glucocorticoid doses higher than any other trial in this review, with the exception of the previously mentioned randomized controlled trial in CNS neoplasms. However, glucocorticoid administration was continuous in these two trials. Also, administration was intravenous; the bioavailability of oral methyprednisolone is 82% [88]. The two trials are very similar, except that enrolment was limited to females with the second trial [30,39]. With this in mind, a survival meta-analysis was performed, with the results presented in Figure 5. Figure 5 shows that this high dose continuous gluccorticoid schedule had a detrimental effect on mortality. In the first trial, the cause for the difference in mortality was unknown. In the second trial, there were significantly more gastrointestinal and cardiovascular adverse events in the glucocorticoid arm; the severity and outcome of these events did not significantly differ. Infectious complications occurred in 11.8% of the treated patients and 14.8% of the placebo patients [39].

The third trial consists mostly of GI patients, and compared indomethacin to prednisolone to placebo. The results suggest that prednisolone might have a beneficial effect on survival [14].

The fourth trial compares a progestational agent to dexamethasone to an androgen; no difference in survival was observed. The patients were mostly lung and GI cancer patients. Fifty three percent and twenty percent of the patients had planned concurrent chemotherapy and radiation therapy respectively.

The fifth trial compares opioids to opioids plus dexamethasone; no survival difference was found. This was a group of patients with very advanced cancer; no patient lived longer than 45 days [61].

There is a meta-analysis of three small RCTs of glucocorticoids in patients with bowel obstruction secondary to malignancy. The treatment period in these 3 trials lasted from 3–10 days; after the treatment period, glucocorticoids were not prohibited. There was no survival difference [62].

There are two trials of chemotherapy +/- glucocorticoids. In the older of these two trials, glucocorticoids had a neutral effect. In this trial, the remission criteria are not readily comparable to those presently used [27]. In the second trial, glucocorticoids ameliorated the GI toxicity of perifosine resulting in longer duration of treatment in the glucocorticoid arm: glucocorticoids may have acted as a chemoprotective agent [52].

Finally, there is one trial of radiation +/- glucocorticoids in patients with epidural metastases of a carcinoma compressing the spinal cord or cauda equina [29]; there is a second trial comparing two doses of glucocorticoids in patients with spinal cord compression [57]. In both of these trials, a large proportion of patients had breast or prostate cancer. There was no survival difference in either trial.

About the quality of the meta-analysis, the inclusion criteria were objective and explicit. The literature search was thorough. There was quality assessment of the clinical trials that made up the meta-analysis. There was independent data abstraction by two reviewers. Individual patient data was used to obtain survival analysis. However, the meta-analysis is based on the results of 83 patients [62].

A description of these trials and the meta-analysis is given in Table 11.

**Table 11:** Randomized Controlled Trials of Glucocorticoids and Meta-analysis in Patients with a Variety of Cancers

Author [reference], Year	Patient Characteristics	Treatment Arms Patient Number (Randomized/Evaluable)	Quality Score	Outcome(s)
Della Cuna et al [30], 1989	preterminal carcinoma; male (196): 38.8% lung, 12.8% stomach, 11.2% buccal cavity and pharynx, 7.1% prostate, 5.1% rectum/rectosigmoid junction female (207): 34.8% breast, 10.1% stomach, 11.1% large intestine, 7.7% cervix uteri, 5.3% lung, 5.3% rectum/rectosigmoid junction	methylprednisolone 125 mg IV daily for 8 weeks (207/NR) vs. placebo (196/NR)	5	mortality at 8 weeks: males 40.2% vs. 35.5%, females 40.0% vs. 27.7% (p < 0.01)
Popiela et al [39], 1989	terminal cancer, female, 85% with gastrointestinal or breast or genitourinary cancers, only solid tumors	methylprednisolone 125 mg IV daily for 56 days (85/NR) vs. placebo (88/NR)	5	mortality at 56 days of 38% vs. 30% (p > 0.05)
Lundholm et al [14], 1994	liver/pancreas 44, colorectal 30, gastric 18, esophagus 15, melanoma 7, breast 3, head and neck 3, miscellaneous 15	indomethacin 50 mg po twice daily (45/NR) vs. prednisolone 10 mg po twice daily (45/NR) vs placebo (45/NR); all treatments until death	2	when all 3 groups compared simultaneously, survival of indomethacin > prednisone > placebo (p < 0.07)
Loprinzi et al [49], 1999	40% lung cancer, 36% GI cancer, no breast or prostate or ovarian or endometrial cancer;	megestrol acetate 800 mg po every day (NR/158) vs. dexamethasone 0.75 mg po four times daily (NR/158) vs. fluoxymesterone 10 mg po twice daily (NR/159)	2	median survival of 126 days with no statistically significant difference between the 3 arms
Mercadante et al [61], 2007	advanced cancer patients on strong opioids	opioids vs. opioids + dexamethasone 8 mg po daily; 76 randomized/66 evaluable	2	Mean survival (range) of 33 (26-40) vs. 37 (28-45) days
Feuer et al [62], 1999, Metaanalysis	patients with bowel obstruction due to malignancy; vast majority either gynecological or GI cancer	glucocorticoids (65/54) vs. no glucocorticoids (32/29)		Kaplan-Meier survival curves and 1 month survival: no differences of statistical significance
Horton et al [27], 1966	19 colorectal cancers, 13 adenocarcinomas of other primary sites, 10 miscellaneous cancers	5-FU IV 15 mg/kg on 1^st^, 2^nd^, 3^rd^, 4^th ^and 6^th ^days and 7.5 mg/kg every other day thereafter until diarrhea, stomatitis or leukopenia (21/NR) vs. same + methyprednisolone 24 mg po daily (21/NR)	4	response rates of 24% vs. 19%
Bernhardt et al [52], 2006	cancers of lung (9), prostate (7), pancreas (3), ovary (2), breast (1), other (4)	docetaxel 75 mg/m^2 ^day 8 of each 21 day cycle + perifosine 50 mg po 1 or 2 or 3 times a day in successive cohorts on days 1-14 vs. same + prednisone 5 mg twice daily	1	2 SD (2 prostate) in 7 evaluable patients vs. 3 PR (1 lung and 2 prostate) and 1 SD (prostate) in 9 evaluable patients
Sorensen et al [29], 1994	patients with compression of spinal cord or cauda equina due to cancer: 34 breast cancers, 6 GI cancer, 5 prostate cancer, 3 lung cancer, 4 sarcoma, 2 melanoma, 1 each of kidney, mesothelioma and thyroid	radiation (30/30) vs. radiation + initial dose of dexamethasone 96 mg IV followed by dexamethasone 96 mg po daily for 3 days followed by 10 day taper (29/27)	2	median survival of 6 months in both arms
Graham et al, [57], 2006	patients with spinal cord compression not due to lymphoma or myeloma: 11 breast or prostate cancer, 9 lung or GI or renal or other	radiation + 16 mg of dexamethasone intravenously for 3 days followed by 13 day taper (9/9) vs. same except 96 mg of dexamethasone for first 3 days (11/11)	1	median survivals of 2.4 vs. 2.1 months

### Randomized Controlled Trials of Glucocorticoids in Lung Cancer

There are two randomized controlled trials of glucocorticoid monotherapy in lung cancer [10,41,42], with both being described in Table 12. In the first trial, those in the glucocorticoid arm did worse than those in the placebo arm. As causes of death, rates of pulmonary infection, hemorrhage, heart failure and perforated ulcer were very similar between the placebo and cortisone arms. The authors stated that they were unable to explain why the cortisone arm did worse [41]. In a later publication regarding this trial, it is noted that results were not different between patients, who had received prior to the trial, surgery, radiation therapy or who hadn't received either therapy. Although it is unclear how systematically this was studied, the authors did not observe a difference in the rate of cancer progression between the placebo and cortisone arms based on x-ray and autopsy information [42]. The second trial is a comparison of prednisolone versus megestrol acetate; those in the megestrol acetate arm did significantly worse [10]. As mentioned in Table 12, no radiation with a curative intent or chemotherapy was given in the second trial (F. Macbeth, personal communication).

**Table 12:** Randomized Controlled Trials of Glucocorticoids in Lung Cancer

Author [reference], Year	Patient Characteristics	Treatment Arms Patient Number (Randomized/Evaluable)	Quality Score	Outcome(s)
Wolf et al [41, 42], 1960	lung carcinoma not amenable to surgery or radiation therapy; distribution of the 4 tumor types (squamous, small cell, adenocarcinoma, large cell) between treatment groups did not statistically differ	Nitrogen mustard 0. 4 mg/kg IV days 1 and 42 (80/70) vs. testosterone propionate 100 mg IM three times weekly (106/99) vs. cortisone 100 mg po daily (86/78) vs. progesterone 2 mg po daily (91/83) vs. diethylstilbestrol 10 mg po daily (89/82) vs. placebo po daily (88/84); all treatments (except for nitrogen mustard) for 12 weeks followed by 8 week taper for oral treatments	4	median survival (days) of 121 vs. 78 vs. 56 vs. 60 vs. 75 vs. 93
Macbeth et al [10], 1994	nonsmall cell and small cell lung cancer	76/72 to either megestrol acetate 480 mg/m^2 ^or prednisolone 15 mg/m^2 ^for 8 weeks; no radiation with a curative intent or chemotherapy given	2	survival worse in the megestrol acetate arm (p = 0.02)
Thatcher et al [34], 1982	metastatic nonsmall cell and small cell lung cancer; no previous chemotherapy or steroids	Cyclophosphamide IV 1.5 g/m^2^, 2.5 g/m^2^, 3.5 g/m^2 ^each separated by 3 weeks (28/28, 16 SCLC) vs. same + prednisolone 100 mg/m^2 ^orally on day of and day after each chemotherapy (29/29, 16 SCLC)	2	response rates of 57% vs. 24%, median duration of response (weeks) of 15 vs. 11 (p > 0.05), median survival (weeks) of 24 vs. 14 (p = 0.004)
Leggas et al [89], 2005	untreated Stage lV NSCLC	4 cycles of carboplatin AUC 5.5 day 1 and gemcitabine 1000 mg/m^2 ^days 1 and 8 every 21 days; 4 days prior to and the day of each chemotherapy treatment in courses 2-4, dexamethasone 16 mg bid (NR/12) vs. 8 mg bid (NR/12) vs none (NR/6)		8 PR vs. 7 PR vs. 2 PR

In lung cancer, there are two randomized controlled trials of chemotherapy +/- glucocorticoids; both are included in Table 12. The first trial was of cyclophosphamide +/-prednisolone; the addition of prednisolone resulted in a lower response rate and decreased survival. Of the planned cyclophosphamide doses, 88% were administered in the cyclophosphamide arm and 69% were administered in the combined arm. As for treatment delays, 4 patients were delayed 1–2 weeks with 3 of these being in the cyclophosphamide arm. No patient died of treatment in the cyclophosphamide arm, but 3 died of infection in the combined arm. Six patients had infections requiring hospitalization in the cyclophosphamide arm, compared to 2 in the combined arm. There was no significant difference in hematologic toxicity between the 2 arms. There was a trend for less leukopenia in the combined arm. In the cyclophosphamide arm, a leukocyte count of less than 1999 cells/mm^3 ^occurred in 43% of patients in cycle 1, 96% in cycle 2 and 100% in cycle 3. In the combined arm, comparable data is 28% in cycle 1, 60% in cycle 2 and 77% in cycle 3 [34]. In the second trial of chemotherapy +/- glucocorticoids, patients were given four cycles of chemotherapy. Those in the chemotherapy + glucocorticoid arms (cohorts 2 and 3) received dexamethasone with the last three cycles; cohort 1 received only chemotherapy. Neutropenia and thrombocytopenia were significantly decreased by the use of glucocorticoids [89].

## Discussion

As monotherapy, glucocorticoids are thought to have activity in breast cancer, prostate cancer and thymoma. The evidence found in this review is consistent with that. In Table 2, no responses were observed in case series data. Although the quality of this generally older data is not comparable to that from present clinical trials, it is unlikely that major changes went undetected. In Table 3, there is mention of responses in cancers other than breast, prostate or thymoma in clinical trial data. However, responses in these older trials were determined by physical examination and xrays. If present day imaging had been used in these trials, it would not have been surprising if response rates were lower than those given. In one trial, it was postulated that some tumor responses may have been a consequence of anti-inflammatory activity, rather than anti-tumor activity [64].

A postulated mechanism of action of glucocorticoids in prostate cancer is adrenal androgen suppression [90]. For breast cancer, postulated mechanisms are adrenocortical inhibition and interaction with glucocorticoid receptors [91]. The one breast cancer trial which looked at whether there was a correlation between ER status and the effect of glucocorticoids did not find one [13]. This lack of correlation suggests that glucocorticoid therapy does not act through the ER receptor. If this is true, a patient's menstrual status may be important with regards to the effectiveness of glucocorticoids. This is relevant, because some of the RCTs of glucocorticoids in breast cancer, especially the chemotherapy trials, included premenopausal women.

Meta-analyses of endocrine therapy +/- glucocorticoids in advanced breast cancer were undertaken. An increased response rate was noted, but there was no improvement in survival. A previous meta-analysis of tamoxifen +/- other endocrine therapy in metastatic breast cancer has been published [92]. This meta-analysis included two of the four papers used in the meta-analysis presented in this publication. The previously published meta-analysis also showed an improved response rate, but no improvement in survival

There was heterogeneity noted in the survival meta-analysis of endocrine therapy +/- glucocorticoids in advanced breast cancer. A critical variable in response to endocrine therapy is ER/PR status. In the four older trials used in that meta-analysis, one trial enrolled patients regardless of ER/PR status [24]. In all four trials, patients of unknown receptor status were enrolled; the percentage of patients with unknown receptor status was as high as 47% [37]. Receptor heterogeneity may explain in part the observed statistical heterogeneity.

Meta-analyses of chemotherapy +/- glucocorticoids in advanced breast cancer were undertaken. Once again, an increased response rate was noted, but no improvement in survival. The meta-analysis of tamoxifen +/- other endocrine therapy in metastatic breast cancer also included a meta-analysis of chemotherapy +/- endocrine therapy. That meta-analysis did not include any of the six trials included in this publication's meta-analysis of chemotherapy +/- glucocorticoids. The meta-analysis of chemotherapy +/- endocrine therapy similarly showed an improved response rate, but no change in survival [92].

In several breast cancer trials of chemotherapy +/- glucocorticoids, there was increased chemotherapy dose administered and less hematologic toxicity in the arms receiving glucocorticoids. The information presented in the results section may underestimate these effects, as trials in which there were nonsignificant differences are not mentioned. This decreased hematologic toxicity is consistent with the known effects of glucocorticoids on the hematopoeitic system [93]. In the RCTs of chemotherapy +/- glucocorticoids of breast cancer in the adjuvant setting, increased chemotherapy dose/dose intensity and decreased hematologic toxicity did not translate into improved outcomes. However, the two trials reporting this were trials of 12 months of chemotherapy [12,85]. With such prolonged chemotherapy, cumulative dose and dose intensity may be less important. Glucocorticoids are commonly given with chemotherapy to patients with metastatic breast cancer as antiemetics; they may be doing more than preventing nausea and vomiting in some patients.

In prostate cancer, glucocorticoids are used in combination with other endocrine therapy (ketoconazole) and chemotherapy. The evidence for this is not strong. If the breast cancer results are applicable to prostate cancer, it is debatable whether such combination therapy is of benefit.

The RCTs of glucocorticoids in GI cancer would suggest that the effect of glucocorticoids is neutral. However, GI cancer includes a diverse group of cancers; one cannot exclude the possibility that glucocorticoids might not have a neutral effect in a particular type of GI cancer. The trial of Lundholm et al is presented in Table 11 because patients with a variety of cancer were eligible; however, 79% of the patients had GI cancer. In that trial, there is a suggestion that glucocorticoids might improve outcome [14].

There were two trials of high dose continuous glucocorticoids compared to placebo in patients with nonhematologic malignancy. There was only one other randomized controlled trial which used a glucocorticoid dose that was greater than the dose used in those two trials. In that trial, patients were on glucocorticoids only one quarter of the time [23]. When the results of the two trials of high dose continous glucocorticoids were combined in a meta-analysis, there was a detrimental effect of glucocorticoids on mortality. This raises the possibility that glucocorticoids, at sufficient dose, may have an adverse effect on patients with nonhematologic malignancy. This is especially relevant to patients with primary and secondary CNS malignancy, where doses of 16 mg per day of dexamethasone are used.

It also raises the question of what is a safe dose of glucocorticoids in patients with nonhematologic malignancy. The answer to this may in part depend on the site of origin of the cancer. In GI cancer, dexamethasone 6 mg daily appears to be safe [26]. In lung cancer, 100 mg daily of cortisone may be unsafe [41]. In the two trials of continuous high dose glucocorticoids, a considerable portion of the patients had breast cancer. In one of the two trials, survival was analyzed according to the origin of the cancer. When the treatment and placebo groups were compared in these analyses, no differences of statistical significance were observed [39].

The only other evidence of glucocorticoids, resulting in a worse outcome than those in the placebo/best supportive care arm, is in the single trial of lung cancer. This 1960 publication gives limited statistical detail. However, it appears that the difference between the glucocorticoid arm and the placebo arm had a p value of 0.02. It is difficult to explain the worse outcome on the dose of glucocorticoid given, as the daily dose was 100 mg of cortisone [41].

The only trial of chemotherapy +/- glucocorticoids, in which the glucocorticoid arm did worse, is in lung cancer. There was a nonsignificant trend for decreased leukopenia in this trial [34]. Others have observed decreased granulocytopenia [94,95] and decreased thrombocytopenia [94] when glucocorticoids are given with chemotherapy in lung cancer patients. In the lung cancer trial of cyclophosphamide +/- prednisone, the authors postulated that the differences found between the 2 arms might be due to the effect of prednisone on the metabolism of cyclophosphamide; however, they themselves noted that both the animal and human data on this effect are conflicting. In the trials of chemotherapy +/- glucocorticoids in breast cancer, a number of the trials included cyclophosphamide as part of CMF. The dose of glucocorticoids given in the lung cancer trial of chemotherapy +/- glucocorticoids is not greatly different than the dose given commonly as an antiemetic to lung cancer patients on chemotherapy.

It cannot be ruled out that glucocorticoids have an effect on the newer targeted therapies. Glucocorticoids are commonly given with monoclonal antibodies. A postulated mechanism of action of one of those antibodies, trastuzumab, is via antibody-dependent cellular cytotoxicity [96]. There is evidence that glucocorticoid may inhibit antibody-dependent cellular cytotoxicity [97].

There are limitations to the meta-analyses presented in this study. Firstly, there may be unpublished randomized controlled trials that are not included. Secondly, survival was extrapolated from summary graphs and data, and the point estimates are not as accurate as those that could have been derived from the individual patient data. Thirdly, the number of trials and the number of patients in each trial tended to be small; this makes heterogeneity more likely. For each meta-analysis, a chi-square test was used to assess heterogeneity. However, a chi-square test has low power if there are few trials or sample sizes are small.

There is evidence that glucocorticoids have an effect on the natural history of some nonhematologic malignancies. However, that is not the only source of glucocorticoids that a cancer is exposed to. One's own body synthesizes endogenous glucocorticoids. This raises the question of what effect endogenous glucocorticoids have on nonhematologic malignancy. The possible effect of endogenous glucocorticoids on lung cancer is the subject of another review (manuscript in preparation).

Breast cancer, prostate cancer and possibly lung cancer show sensitivity to glucocorticoids. There may be variability within cancers of each type in their sensitivity. This raises the issue of predictive factors, that might assist in assessing glucocorticoid sensitivity. Glucocorticoid receptor status or histologic subtype (in lung cancer) might be relevant. A genomic/proteomic approach to explore this possibility might be useful.

## Conclusion

The effect of glucocorticoids in nonhematologic malignancy depend on the primary tumor site. Glucocorticoids have a beneficial effect in breast and prostate cancer as monotherapy. In combination with chemotherapy or other endocrine therapy in breast cancer; glucocorticoids increase response rate, but do not change survival. In GI cancer, they most likely have a neutral effect. High dose continuous glucocorticoids, in patients with nonhematologic malignancy, decrease survival. Such treatment should be avoided in patients with nonhematologic malignancy. Based on ASCO criteria, this is a grade B recommendation based on level ll evidence. In lung cancer, glucocorticoids might have a deleterious effect by themselves and when given with chemotherapy. It is recommended that glucocorticoid use in lung cancer patients be kept to the minimum required. This is a grade B recommendation based on level ll evidence.

## List of Abbreviations

AV: adriamycin 60 mg/m^2 ^IV day 1 and vincristine 1.2 mg/m^2 ^IV day 1 every 21 days, BCNU: carmustine, CI: confidence interval, CMF: cyclophosphamide 100 mg/m^2 ^po daily days 1–14 and methotrexate 40 mg/m^2 ^IV days 1 and 8 and 5-FU 600 mg/m^2 ^IV days 1 and 8 every 28 days, CMFP: CMF and prednisone 40 mg/m^2^/day days 1–14, CR: complete response, ER: estrogen receptor, 5-FU: 5-fluorouracil, FUDR: fluorodeoxyuridine, IM: intramuscular, IV: intravenous, m^2^: meters squared, MPA: medroxyprogesterone acetate, -ve: negative, ns: not significant, NR: not reported, NSC-17256: 6α-methylpreg-4-ene-3,11,20-trione, NSCLC: nonsmall cell lung cancer, PD: progressive disease, +ve: positive, PR: partial response, PgR: progesterone receptor, po: by mouth, SCLC: small cell lung cancer, SD: stable disease, SE: standard error, vs.: versus

## Competing interests

The author(s) declare that they have no competing interests.

## Pre-publication history

The pre-publication history for this paper can be accessed here:



## Supplementary Material

Additional file 1 Search strategies. Provides the search strategies for PubMed, EMBASE, the Cochrane Library (including ACP Journal Club) and CINAHL. Click here for file
