# Supplementary material for: Systematic review of the clinical effect of glucocorticoids on nonhematologic malignancy
Source: BMC Cancer. 2008 Mar 28;8:84. doi: 10.1186/1471-2407-8-84 (PMC2330150; doi:10.1186/1471-2407-8-84)
Supplement: Additional file 1 — Search strategies. Provides the search strategies for PubMed, EMBASE, the Cochrane Library (including ACP Journal Club) and CINAHL. [file 1471-2407-8-84-S1.doc]

**Search Strategies**

Search strategies for PubMed, EMBASE, the Cochrane Library and CINAHL are presented. For Pubmed and EMBASE, the searches for glucocorticoids and non-hematologic malignancy were combined with each of the subsequent searches. For the Cochrane Library and CINAHL, the searches for glucocorticoids and non-hematologic malignancy were combined.

1)*PubMed Searches*

Glucocorticoid Search

"Pregnadienediols"[MeSH] OR hydrocortisone OR corticosterone OR cortisone OR glucocorticoid* OR "Pregnadienetriols"[MeSH] OR dexamethasone OR prednisone OR prednisolone OR methylprednisolone OR corticosteroid*

Non-Hematologic Malignancy Search

("Neoplasms"[MeSH] OR cancer) NOT "Histiocytic Disorders, Malignant"[MAJR] NOT "Leukemia"[MAJR] NOT "Lymphatic Vessel Tumors"[MAJR] NOT "Lymphoma"[MAJR] NOT "Hematologic Neoplasms"[MAJR]

Randomized Controlled Trial Search (from Cochrane Handbook Search)

randomized controlled trial[pt] OR controlled clinical trial[pt] OR randomized controlled trials[mh] OR random allocation [mh] OR double-blind method[mh] OR single-blind method[mh] OR clinical trial[pt] OR clinical trials[mh] OR ("clinical trial"[tw]) OR ((singl*[tw] OR doubl*[tw] OR trebl*[tw] OR tripl*[tw]) AND (mask*[tw] OR blind*[tw])) OR (placebos[mh] OR placebo*[tw] OR random*[tw] OR research design[mh:noexp] OR comparative study [pt] OR evaluation studies[mh] OR follow-up studies [mh] OR prospective studies[mh] OR control*[tw] OR prospectiv*[tw] OR volunteer*[tw]) NOT (animals[mh] NOT human[mh])

Phase 1/ll Clinical Trial Search

phase I OR phase II

Review (Systematic or Non-Systematic) Search

review* OR overview*

Meta-Analysis Search

"Meta-Analysis"[Publication Type] OR meta-anal* OR metaanaly* OR meta analy*

Other Clinical Observational Studies Search

"Case-Control Studies"[MeSH] OR "Cohort Studies"[MeSH] OR ("Comparative Study"[MeSH] OR "Comparative Study"[Publication Type]) OR "Follow-Up Studies"[MeSH] OR "Prospective Studies"[MeSH] OR "Retrospective Studies"[MeSH] OR "Time Factors"[MeSH] OR "Treatment Outcome"[MeSH] OR "Case Reports"[Publication Type] OR "Clinical Trial"[Publication Type] OR "Evaluation Studies"[Publication Type] OR case series OR cases OR chang* OR cohort* OR compare* OR compara* OR consecutive* OR evaluat* OR follow* OR non compara* OR noncompara* OR non random* OR nonrandom* OR observational OR prospective* OR retrospective* OR reviewed OR case control

Practice Guidelines Search

"Practice Guidelines"[MeSH] OR "Practice Guideline"[Publication Type]

2)*Embase Searches*

Glucocorticoid Search

exp glucocorticoid/ or glucocorticoid.mp. or corticosteroid.mp. or pregnane derivative.mp. or cortisone.tw. or hydrocortisone.tw. or dexamethasone.tw. or prednisone.tw. or prednisolone.tw. or methyprednisolone.tw.

Non-Hematologic Malignancy Search

(exp neoplasm/ or cancer.mp.) not exp *leukemia/ not exp *lymphatic leukemia/ not exp *malignant plasmacytoma/

Randomized Controlled Trial Search

randomized controlled trial/ or controlled study/ or randomization/ or double blind procedure/ or single blind procedure/ or clinical trial/ or clinical trial.tw. or (( singl$ or doubl$ or trebl$ or tripl$) and (mask$ or blind$)).tw. or placebo/ or placebo$.tw. or random$.tw. or comparative study/ or evaluation/ or follow up/ or prospective study/ or control$.tw. or prospectiv$.tw. or volunteer$.tw. or methodology/

Phase 1 Clinical Trial Search

phase 1 clinical trial.mp.

Phase 2 Clinical Trial Search

phase 2 clinical trial.mp.

Review (Systematic or Non-Systematic) Search

(review$ or overview$).mp.

Meta Analysis Search

(meta-analysis or meta-anal$ or metaanaly$ or meta analy$).mp.

Other Clinical Observational Studies Search

case report/ or clinical trial/ or cohort analysis/ or comparative study/ or controlled study/ or follow up/ or major clinical study/ or prospective study/ or retrospective study/ or treatment outcome/ or baseline.tw. or case control$.tw. or case series.tw. or cases.tw. or chang$.tw. or consecutive$.tw. or evaluat$.tw. or non compara$.tw. or noncompara$.tw. or non random$.tw. or nonrandom$.tw. or observational.tw. or reviewed.tw. or cohort$.mp. or compar$.mp. or follow$.mp. or prospective$.mp. or retrospective$.mp.

Practice Guideline Search

exp practice guideline/ or practice guideline.mp.

3)*Cochrane Searches*

A. CCTR

Non-Hematologic Malignancy Search

(cancer.mp. or exp neoplasms/) not exp *leukemia/ not exp *lymphatic vessel tumors/ not exp *lymphoma/ not exp *hematologic neoplasms/

Glucocorticoid Search

(prednisolone or fluorometholone or dexamethasone).mp. or exp dexamethasone/ or prednisone.mp. or cortisone.mp. or fluorometholone.mp. or hydrocortisone.mp. or corticosterone.mp. or methylprednisolone.mp. or exp methylprednisolone/

B. ACP Journal Club/DARE/Cochrane Database of Systematic Reviews

Non-Hematologic Malignancy Search

(neoplasms or neoplasm or cancer).mp.

Glucocorticoid Search

(prednisolone or fluorometholone or dexamethasone or prednisone or cortisone or hydrocortisone or corticosterone or methylprednisolone).mp.

4.*CINAHL*

Non-Hematologic Malignancy Search

Neoplasm/ or cancer.mp.

Glucocorticoid Search

Dexamethasone.mp. or cortisone.mp. or hydrocortisone.mp. or prednisone.mp. or prednisolone.mp. or methylprednisolone.mp. or glucorticoid$.mp.
